# (2-Methyl-1-phenyl­sulfonyl-1*H*-indol-3-yl)methanol

**DOI:** 10.1107/S1600536808003024

**Published:** 2008-01-30

**Authors:** G. Chakkaravarthi, V. Dhayalan, A. K. Mohanakrishnan, V. Manivannan

**Affiliations:** a Department of Physics, CPCL Polytechnic College, Chennai 600 068, India; b Department of Organic Chemistry, University of Madras, Guindy Campus, Chennai 600 025, India; c Department of Physics, Presidency College, Chennai 600 005, India

## Abstract

In the title compound, C_16_H_15_NO_3_S, the plane of the phenyl ring forms a dihedral angle of 80.37 (8)° with the indole ring system. The crystal packing is stabilized by weak O—H⋯O hydrogen bonds which link the mol­ecules into infinite chains along the *a* axis of the crystal.

## Related literature

For biological activity, see: Nieto *et al.* (2005 ▶); Pomarnacka & Kozlarska-Kedra (2003 ▶). For the structure of closely related compounds, see: Chakkaravarthi *et al.* (2007 ▶); Liu *et al.* (2007 ▶).
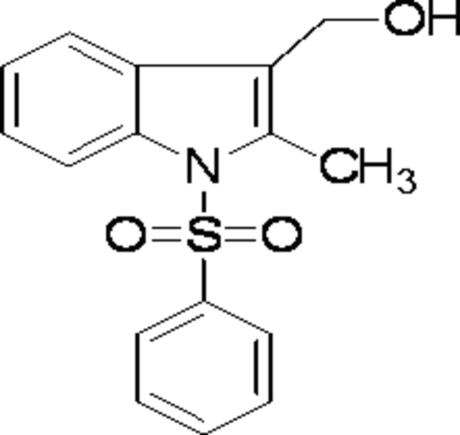


## Experimental

### 

#### Crystal data

C_16_H_15_NO_3_S
*M* *_r_* = 301.35
Triclinic, 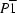

*a* = 8.3780 (4) Å
*b* = 9.6969 (5) Å
*c* = 9.9630 (4) Å
α = 78.718 (2)°
β = 65.347 (3)°
γ = 78.884 (2)°
*V* = 715.77 (6) Å^3^
*Z* = 2
Mo *K*α radiation
μ = 0.23 mm^−1^
*T* = 295 (2) K
0.22 × 0.18 × 0.16 mm

#### Data collection

Bruker Kappa APEXII diffractometer
Absorption correction: multi-scan (*SADABS*; Sheldrick, 1996 ▶) *T*_min_ = 0.920, *T*_max_ = 0.963
14857 measured reflections
4088 independent reflections
3267 reflections with *I* > 2σ(*I*)
*R*_int_ = 0.021

#### Refinement

*R*[*F* ^2^ > 2σ(*F* ^2^)] = 0.058
*wR*(*F* ^2^) = 0.241
*S* = 1.05
4088 reflections
191 parameters
H-atom parameters constrained
Δρ_max_ = 0.43 e Å^−3^
Δρ_min_ = −0.48 e Å^−3^

### 

Data collection: *APEX2* (Bruker, 2004 ▶); cell refinement: *APEX2*; data reduction: *APEX2*; program(s) used to solve structure: *SHELXS97* (Sheldrick, 2008 ▶); program(s) used to refine structure: *SHELXL97* (Sheldrick, 2008 ▶); molecular graphics: *PLATON* (Spek, 2003 ▶); software used to prepare material for publication: *SHELXL97*.

## Supplementary Material

Crystal structure: contains datablocks I, global. DOI: 10.1107/S1600536808003024/ya2066sup1.cif

Structure factors: contains datablocks I. DOI: 10.1107/S1600536808003024/ya2066Isup2.hkl

Additional supplementary materials:  crystallographic information; 3D view; checkCIF report

## Figures and Tables

**Table 1:** Hydrogen-bond geometry (Å, °)

*D*—H⋯*A*	*D*—H	H⋯*A*	*D*⋯*A*	*D*—H⋯*A*
O3—H3⋯O2^i^	0.82	2.59	3.276 (5)	142

Symmetry code: (i) 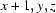.

**Fig. 1.**
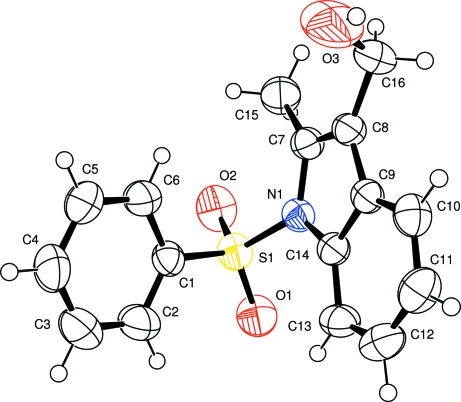
The molecular structure of (I), with atom labels and 50% probability displacement ellipsoids; H atoms are shown as small spheres of arbitrary radius.

**Fig. 2.**
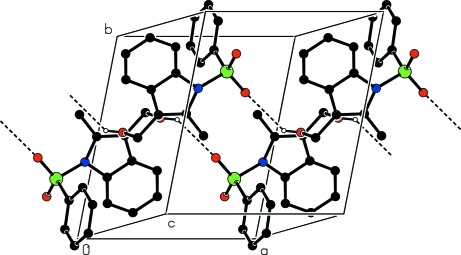
The packing of (I), viewed down the c axis. H-bonds are shown as dashed lines; H atoms not involved in hydrogen bonding have been omitted.

## supplementary crystallographic information

### Comment

In continuation of our studies of benzenesulfonamide derivatives, which are known to exhibit antibacterial (Nieto *et al.*, 2005), anticancer and anti - HIV (Pomarnacka & Kozlarska-Kedra, 2003) activities, we determined the crystal structure of the title compound, (I). The geometric parameters of the molecule of (I) (Fig. 1) agree well with those reported for similar structures (Chakkaravarthi *et al.*, 2007; Liu *et al.*, 2007).

The plane of the phenyl ring forms the dihedral angle of 80.37 (8)° with the indole ring system. The N1—S1—C1 plane is also approximately orthogonal to indole (dihedral angle 79.21 (6)°) and makes an angle of 57.86 (11)° with the phenyl plane.

The crystal packing of (I) is stabilized by a rather weak O—H···O bonds which link the molecules into the infinite chains along the *x*-axis of the crystal (Fig. 2, Table 2).

### Experimental

Benzenesulfonyl chloride (7.75 ml, 43.9 mmol), 60% NaOH solution (60 g in 100 ml), along with tetrabutylammonium hydrogensulfate (1.5 g) were added to the solution of 2-methylindole-3-carboxaldehyde (8.0 g, 50.3 mmol) in distilled benzene (200 ml). The two-phase system thus formed was stirred at room temperature for 2 h. It was then diluted with water (200 ml) and the organic layer was separated. The aqueous layer was extracted with benzene (two times by 30 ml). The combined organic layer was dried (Na_2_SO_4_). The benzene was then completely removed and the crude product was recrystallized from methanol to get 1-phenylsulfonyl-2-methylindole-3-carboxaldehyde.

NaBH_4_ (2.97 g, 78.60 mmol) was added slowly to a solution of 1-phenylsulfonyl-2-methylindole-3-carboxaldehyde (3 g, 13.10 mmol) in THF (30 ml). The reaction mixture was stirred at room temperature for 3 hrs. Dilute HCl (10%) was cautiously added until the solution became acidic. After most of the THF was removed *in vacuo*, the solution was extracted with dichloromethane and dried (MgSO_4_); the solvent was then removed under vacuo. The crude product thus obtained was recrystallized from 10% ethyl acetate/n-hexane.

### Refinement

H atoms were positioned geometrically and refined using riding model approximation with C—H = 0.93 Å and *U*_iso_(H) = 1.2Ueq(C) for aromatic C—H, C—H = 0.97 Å and *U*_iso_(H) = 1.2Ueq(C) for CH_2_, C—H = 0.96 Å and *U*_iso_(H) = 1.5Ueq(C) for CH_3_ and O—H = 0.82 Å and *U*_iso_(H) = 1.5Ueq(O) for OH. The hydroxyl O3 atom showed rather high thermal displacement parameters, however the attempts to introduce an alternative position and refine disordered model proved unsuccessful.

### Crystal data

**Table:**

C_16_H_15_NO_3_S	*Z* = 2
*M**_r_* = 301.35	*F*_000_ = 316
Triclinic, *P*1	*D*_x_ = 1.398 Mg m^−^^3^
Hall symbol: -P 1	Mo *K*α radiation λ = 0.71073 Å
*a* = 8.3780 (4) Å	Cell parameters from 8443 reflections
*b* = 9.6969 (5) Å	θ = 2.7–26.2º
*c* = 9.9630 (4) Å	µ = 0.23 mm^−^^1^
α = 78.718 (2)º	*T* = 295 (2) K
β = 65.347 (3)º	Block, colourless
γ = 78.884 (2)º	0.22 × 0.18 × 0.16 mm
*V* = 715.77 (6) Å^3^	

### Data collection

**Table:**

Bruker Kappa APEXII diffractometer	4088 independent reflections
Radiation source: fine-focus sealed tube	3267 reflections with *I* > 2σ(*I*)
Monochromator: graphite	*R*_int_ = 0.021
*T* = 295(2) K	θ_max_ = 30.0º
ω and φ scans	θ_min_ = 2.2º
Absorption correction: multi-scan(SADABS; Sheldrick, 1996)	*h* = −11→11
*T*_min_ = 0.920, *T*_max_ = 0.963	*k* = −13→13
14857 measured reflections	*l* = −14→14

### Refinement

**Table:**

Refinement on *F*^2^	Secondary atom site location: difference Fourier map
Least-squares matrix: full	Hydrogen site location: inferred from neighbouring sites
*R*[*F*^2^ > 2σ(*F*^2^)] = 0.058	H-atom parameters constrained
*wR*(*F*^2^) = 0.241	*w* = 1/[σ^2^(*F*_o_^2^) + (0.0931*P*)^2^ + 0.2366*P*] where *P* = (*F*_o_^2^ + 2*F*_c_^2^)/3
*S* = 1.05	(Δ/σ)_max_ < 0.001
4088 reflections	Δρ_max_ = 0.43 e Å^−^^3^
191 parameters	Δρ_min_ = −0.48 e Å^−^^3^
Primary atom site location: structure-invariant direct methods	Extinction correction: none

### Fractional atomic coordinates and isotropic or equivalent isotropic displacement parameters (Å^2^)

**Table:**

	*x*	*y*	*z*	*U*_iso_*/*U*_eq_	
S1	0.70206 (7)	0.26671 (6)	0.24015 (6)	0.0505 (2)	
O1	0.7232 (3)	0.1941 (2)	0.1217 (2)	0.0682 (6)	
O2	0.5477 (2)	0.3641 (2)	0.2984 (3)	0.0705 (6)	
O3	1.1581 (6)	0.5512 (4)	0.3706 (4)	0.1316 (14)	
H3	1.2640	0.5450	0.3519	0.197*	
N1	0.8742 (2)	0.35733 (18)	0.17689 (19)	0.0434 (4)	
C1	0.7297 (3)	0.1424 (2)	0.3853 (2)	0.0464 (4)	
C2	0.7397 (5)	0.0001 (3)	0.3817 (4)	0.0705 (8)	
H2	0.7350	−0.0317	0.3013	0.085*	
C3	0.7567 (6)	−0.0952 (3)	0.4989 (4)	0.0831 (10)	
H3A	0.7620	−0.1917	0.4982	0.100*	
C4	0.7657 (4)	−0.0484 (3)	0.6162 (3)	0.0709 (8)	
H4	0.7779	−0.1133	0.6946	0.085*	
C5	0.7569 (5)	0.0929 (3)	0.6185 (3)	0.0705 (8)	
H5	0.7647	0.1236	0.6981	0.085*	
C6	0.7364 (4)	0.1917 (3)	0.5037 (3)	0.0594 (6)	
H6	0.7275	0.2883	0.5064	0.071*	
C7	0.8842 (3)	0.4756 (2)	0.2371 (2)	0.0449 (4)	
C8	1.0560 (3)	0.4853 (2)	0.2018 (2)	0.0453 (4)	
C9	1.1626 (3)	0.3694 (2)	0.1214 (2)	0.0413 (4)	
C10	1.3442 (3)	0.3292 (3)	0.0597 (3)	0.0537 (5)	
H10	1.4219	0.3825	0.0652	0.064*	
C11	1.4065 (4)	0.2092 (3)	−0.0093 (3)	0.0594 (6)	
H11	1.5275	0.1794	−0.0481	0.071*	
C12	1.2929 (4)	0.1321 (3)	−0.0224 (3)	0.0615 (6)	
H12	1.3393	0.0523	−0.0718	0.074*	
C13	1.1125 (4)	0.1701 (2)	0.0358 (3)	0.0525 (5)	
H13	1.0363	0.1171	0.0277	0.063*	
C14	1.0485 (3)	0.2911 (2)	0.1073 (2)	0.0400 (4)	
C15	0.7267 (4)	0.5750 (3)	0.3152 (3)	0.0650 (7)	
H15A	0.6612	0.5303	0.4133	0.097*	
H15B	0.6525	0.5993	0.2601	0.097*	
H15C	0.7648	0.6594	0.3226	0.097*	
C16	1.1300 (4)	0.5945 (3)	0.2393 (3)	0.0649 (7)	
H16A	1.2413	0.6143	0.1580	0.078*	
H16B	1.0488	0.6816	0.2494	0.078*	

### Atomic displacement parameters (Å^2^)

**Table:**

	*U*^11^	*U*^22^	*U*^33^	*U*^12^	*U*^13^	*U*^23^
S1	0.0467 (3)	0.0609 (4)	0.0548 (4)	−0.0134 (2)	−0.0299 (3)	−0.0024 (2)
O1	0.0776 (13)	0.0891 (14)	0.0633 (11)	−0.0306 (11)	−0.0455 (10)	−0.0057 (10)
O2	0.0440 (9)	0.0813 (13)	0.0859 (14)	−0.0033 (9)	−0.0311 (9)	−0.0021 (11)
O3	0.224 (4)	0.128 (3)	0.104 (2)	−0.087 (3)	−0.104 (2)	0.0019 (19)
N1	0.0453 (9)	0.0437 (8)	0.0451 (9)	−0.0082 (7)	−0.0214 (7)	−0.0037 (6)
C1	0.0469 (10)	0.0499 (10)	0.0458 (10)	−0.0151 (8)	−0.0192 (8)	−0.0027 (8)
C2	0.100 (2)	0.0570 (14)	0.0674 (16)	−0.0225 (14)	−0.0387 (16)	−0.0107 (12)
C3	0.122 (3)	0.0477 (13)	0.081 (2)	−0.0179 (16)	−0.043 (2)	0.0014 (13)
C4	0.085 (2)	0.0636 (15)	0.0549 (14)	−0.0142 (14)	−0.0242 (13)	0.0098 (11)
C5	0.094 (2)	0.0730 (17)	0.0455 (12)	−0.0156 (15)	−0.0283 (13)	−0.0044 (11)
C6	0.0810 (17)	0.0508 (12)	0.0519 (12)	−0.0122 (11)	−0.0300 (12)	−0.0066 (9)
C7	0.0538 (11)	0.0378 (9)	0.0425 (9)	−0.0039 (8)	−0.0198 (8)	−0.0041 (7)
C8	0.0566 (12)	0.0404 (9)	0.0430 (9)	−0.0100 (8)	−0.0218 (9)	−0.0055 (7)
C9	0.0468 (10)	0.0430 (9)	0.0384 (8)	−0.0086 (7)	−0.0207 (8)	−0.0031 (7)
C10	0.0458 (11)	0.0631 (13)	0.0558 (12)	−0.0105 (10)	−0.0229 (10)	−0.0058 (10)
C11	0.0522 (13)	0.0661 (14)	0.0540 (12)	0.0030 (11)	−0.0202 (10)	−0.0070 (11)
C12	0.0697 (16)	0.0582 (13)	0.0569 (13)	0.0096 (11)	−0.0272 (12)	−0.0205 (10)
C13	0.0667 (14)	0.0486 (11)	0.0529 (11)	−0.0072 (10)	−0.0307 (10)	−0.0137 (9)
C14	0.0472 (10)	0.0410 (9)	0.0363 (8)	−0.0073 (7)	−0.0210 (7)	−0.0023 (7)
C15	0.0632 (15)	0.0511 (12)	0.0709 (16)	0.0048 (11)	−0.0186 (12)	−0.0157 (11)
C16	0.0821 (18)	0.0538 (13)	0.0680 (15)	−0.0242 (13)	−0.0294 (13)	−0.0134 (11)

### Geometric parameters (Å, °)

**Table:**

S1—O2	1.421 (2)	C7—C8	1.350 (3)
S1—O1	1.422 (2)	C7—C15	1.491 (3)
S1—N1	1.6619 (18)	C8—C9	1.439 (3)
S1—C1	1.757 (2)	C8—C16	1.499 (3)
O3—C16	1.396 (4)	C9—C10	1.389 (3)
O3—H3	0.8200	C9—C14	1.394 (3)
N1—C14	1.412 (3)	C10—C11	1.371 (4)
N1—C7	1.425 (3)	C10—H10	0.9300
C1—C2	1.373 (4)	C11—C12	1.377 (4)
C1—C6	1.382 (3)	C11—H11	0.9300
C2—C3	1.380 (4)	C12—C13	1.377 (4)
C2—H2	0.9300	C12—H12	0.9300
C3—C4	1.368 (5)	C13—C14	1.391 (3)
C3—H3A	0.9300	C13—H13	0.9300
C4—C5	1.362 (5)	C15—H15A	0.9600
C4—H4	0.9300	C15—H15B	0.9600
C5—C6	1.388 (4)	C15—H15C	0.9600
C5—H5	0.9300	C16—H16A	0.9700
C6—H6	0.9300	C16—H16B	0.9700
			
O2—S1—O1	119.32 (13)	C7—C8—C16	127.6 (2)
O2—S1—N1	106.85 (11)	C9—C8—C16	123.9 (2)
O1—S1—N1	106.16 (11)	C10—C9—C14	119.9 (2)
O2—S1—C1	109.90 (12)	C10—C9—C8	132.5 (2)
O1—S1—C1	109.10 (12)	C14—C9—C8	107.59 (18)
N1—S1—C1	104.41 (9)	C11—C10—C9	118.6 (2)
C16—O3—H3	109.5	C11—C10—H10	120.7
C14—N1—C7	107.57 (17)	C9—C10—H10	120.7
C14—N1—S1	120.92 (14)	C10—C11—C12	121.1 (2)
C7—N1—S1	125.77 (15)	C10—C11—H11	119.4
C2—C1—C6	121.3 (2)	C12—C11—H11	119.4
C2—C1—S1	120.2 (2)	C13—C12—C11	121.6 (2)
C6—C1—S1	118.42 (18)	C13—C12—H12	119.2
C1—C2—C3	119.1 (3)	C11—C12—H12	119.2
C1—C2—H2	120.4	C12—C13—C14	117.4 (2)
C3—C2—H2	120.4	C12—C13—H13	121.3
C4—C3—C2	120.4 (3)	C14—C13—H13	121.3
C4—C3—H3A	119.8	C13—C14—C9	121.3 (2)
C2—C3—H3A	119.8	C13—C14—N1	131.2 (2)
C5—C4—C3	120.1 (3)	C9—C14—N1	107.57 (17)
C5—C4—H4	119.9	C7—C15—H15A	109.5
C3—C4—H4	119.9	C7—C15—H15B	109.5
C4—C5—C6	120.9 (3)	H15A—C15—H15B	109.5
C4—C5—H5	119.5	C7—C15—H15C	109.5
C6—C5—H5	119.5	H15A—C15—H15C	109.5
C1—C6—C5	118.1 (2)	H15B—C15—H15C	109.5
C1—C6—H6	121.0	O3—C16—C8	112.6 (2)
C5—C6—H6	121.0	O3—C16—H16A	109.1
C8—C7—N1	108.74 (19)	C8—C16—H16A	109.1
C8—C7—C15	127.7 (2)	O3—C16—H16B	109.1
N1—C7—C15	123.4 (2)	C8—C16—H16B	109.1
C7—C8—C9	108.49 (19)	H16A—C16—H16B	107.8
			
O2—S1—N1—C14	−178.08 (15)	C15—C7—C8—C9	−177.5 (2)
O1—S1—N1—C14	−49.73 (17)	N1—C7—C8—C16	178.9 (2)
C1—S1—N1—C14	65.49 (17)	C15—C7—C8—C16	3.4 (4)
O2—S1—N1—C7	32.0 (2)	C7—C8—C9—C10	180.0 (2)
O1—S1—N1—C7	160.38 (18)	C16—C8—C9—C10	−0.9 (4)
C1—S1—N1—C7	−84.39 (18)	C7—C8—C9—C14	1.1 (2)
O2—S1—C1—C2	123.0 (3)	C16—C8—C9—C14	−179.8 (2)
O1—S1—C1—C2	−9.6 (3)	C14—C9—C10—C11	−2.3 (3)
N1—S1—C1—C2	−122.7 (2)	C8—C9—C10—C11	178.9 (2)
O2—S1—C1—C6	−55.4 (2)	C9—C10—C11—C12	2.2 (4)
O1—S1—C1—C6	172.0 (2)	C10—C11—C12—C13	−1.4 (4)
N1—S1—C1—C6	58.9 (2)	C11—C12—C13—C14	0.7 (4)
C6—C1—C2—C3	0.0 (5)	C12—C13—C14—C9	−0.9 (3)
S1—C1—C2—C3	−178.3 (3)	C12—C13—C14—N1	179.7 (2)
C1—C2—C3—C4	−0.8 (6)	C10—C9—C14—C13	1.8 (3)
C2—C3—C4—C5	0.4 (6)	C8—C9—C14—C13	−179.15 (18)
C3—C4—C5—C6	0.8 (5)	C10—C9—C14—N1	−178.74 (18)
C2—C1—C6—C5	1.1 (4)	C8—C9—C14—N1	0.3 (2)
S1—C1—C6—C5	179.5 (2)	C7—N1—C14—C13	177.9 (2)
C4—C5—C6—C1	−1.6 (5)	S1—N1—C14—C13	23.2 (3)
C14—N1—C7—C8	2.2 (2)	C7—N1—C14—C9	−1.5 (2)
S1—N1—C7—C8	155.38 (16)	S1—N1—C14—C9	−156.26 (14)
C14—N1—C7—C15	178.0 (2)	C7—C8—C16—O3	93.7 (4)
S1—N1—C7—C15	−28.9 (3)	C9—C8—C16—O3	−85.2 (3)
N1—C7—C8—C9	−2.0 (2)		

### Hydrogen-bond geometry (Å, °)

**Table:**

*D*—H···*A*	*D*—H	H···*A*	*D*···*A*	*D*—H···*A*
O3—H3···O2^i^	0.82	2.59	3.276 (5)	142

Symmetry codes: (i) *x*+1, *y*, *z*.
